# The Usual Suspects: Comparison of the Relative Roles of Potential Urban Chikungunya Virus Vectors in Australia

**DOI:** 10.1371/journal.pone.0134975

**Published:** 2015-08-06

**Authors:** Cassie C. Jansen, Craig R. Williams, Andrew F. van den Hurk

**Affiliations:** 1 Metro North Public Health Unit, Queensland Health, Windsor, Queensland, Australia; 2 Sansom Institute for Health Research, University of South Australia, Adelaide, South Australia, Australia; 3 Forensic and Scientific Services, Department of Health, Queensland Government, Coopers Plains, Queensland, Australia; Centro de Pesquisas René Rachou, BRAZIL

## Abstract

The global re-emergence of chikungunya virus (CHIKV) over the last decade presents a serious public health risk to Australia. An increasing number of imported cases further underline the potential for local transmission to occur if local mosquitoes bite an infected traveller. Laboratory experiments have identified a number of competent Australian mosquito species, including the primary vectors of CHIKV abroad, *Aedes aegypti* and *Aedes albopictus*, and local endemic species *Aedes vigilax* and *Aedes notoscriptus*. The implication of these additional endemic species as potential vectors has generated much uncertainty amongst public health professionals regarding their actual role in CHIKV transmission in the field. Using data estimated from or documented in the literature, we parameterise a simple vectorial capacity model to evaluate the relative roles of Australian mosquito species in potential CHIKV transmission. The model takes into account a number of key biological and ecological variables which influence the role of a species in field transmission, including population density, human feeding rates, mosquito survival rates and vector competence. We confirm the relative importance of *Ae*. *aegypti* and *Ae*. *albopictus* in sustaining potential CHIKV transmission in Australia. Even at maximum estimated densities and human feeding rates, *Ae*. *vigilax* and *Ae*. *notoscriptus* are likely to play a relatively minor role in CHIKV transmission, when compared with either *Ae*. *aegypti* or *Ae*. *albopictus*. This relatively straightforward analysis has application for any region where mosquito species have been incriminated in vector competence experiments, but where their actual role in CHIKV transmission has not been established.

## Introduction

Chikungunya virus (CHIKV) is a mosquito-borne pathogen that has re-emerged globally in the last decade. First isolated in 1952–53 in Tanzania, the name ‘chikungunya’ describes the painful signs associated with the polyarthritic disease syndrome caused by infection with this virus [1]. The virus is transmitted between humans by mosquitoes, particularly *Aedes aegypti* and *Aedes albopictus* in urban settings. Since 2004, CHIKV has spread from East Africa, through the islands of the western Indian Ocean, into the Indian sub-continent and Southeast Asia [2, 3]. In 2007, a traveller from India introduced the virus into north-eastern Italy, causing the first European outbreak of the virus [4]. Emerging for the first time in the Western Hemisphere in late 2013, autochthonous cases have been reported throughout countries in the Caribbean, and in Central and South America [5–8] and, most recently, the first locally transmitted cases in the USA have been recorded in Florida [8, 9]. Outbreaks of CHIKV are often explosive, as evidenced on Reunion Island, where an estimated 244,000 cases represented a prevalence of 35% of the population [10].

Recent outbreaks in nearby locations in the Pacific region including, but not limited to, Papua New Guinea (PNG), New Caledonia, Tonga, Samoa and the Federated states of Micronesia [11–13] have caused considerable concern amongst public health professionals in Australia. Not surprisingly, there have been an increasing number of imported cases into Australia. Between 2002 and 2012, 168 imported cases were reported over the ten-year period [14], but in 2013 alone, 133 cases were notified to the National Notifiable Diseases Surveillance System, followed by 107 in 2014 [15]. If appropriate mosquito vectors are present, local transmission in Australia arising from an imported case is certainly possible, in a scenario similar to what has already occurred in Italy and the Caribbean.

Given this threat, laboratory experiments were undertaken to determine which Australian mosquito species could become infected with and transmit CHIKV [16, 17]. Such physiological competence demonstrates compatibility between a vector species and the virus in question, and is a requisite for a mosquito to be implicated in the transmission of a virus such as CHIKV. These studies demonstrated that a number of endemic species are physiologically competent at becoming infected with, and transmitting CHIKV. Not surprisingly, Australian populations of both *Ae*. *aegypti* and *Ae*. *albopictus* were highly competent. However, the current distribution of these two species in Australia is relatively limited, with *Ae*. *aegypti* occurring predominantly in north Queensland—with isolated populations in central and southern Queensland [18, 19] and *Ae*. *albopictus* present only in the Torres Strait Islands to the north of Cape York [20, 21].

In addition to confirming the vector competence of Australian populations of both *Ae*. *aegypti* and *Ae*. *albopictus*, van den Hurk and others [16] also identified native Australian species, including *Aedes vigilax* and *Aedes notoscriptus* as competent vectors. This has important implications, as these species are geographically widespread, and are abundant in urban areas in both tropical and temperate Australia. However, their potential role in field transmission is uncertain, because the virus has never been isolated from them in nature. Further, species-specific biological traits, such as blood feeding behaviour and relative abundance, provide an additional source of uncertainty when predicting their potential roles in field transmission, and have not been considered in detail. Thus, the potential role of various vector species is prone to misinterpretation when assessing risk and during the development of guidelines for CHIKV risk management.

Herein, we apply a simple vectorial capacity model first developed for malaria vectors [22, 23] to evaluate the relative roles of Australian mosquito species in potential CHIKV transmission. The model takes into account a number of key variables such as density in relation to humans, human feeding rates, mosquito survival rates, the incubation period of the virus in the mosquito and the transmission rate.

## Materials and Methods

### Model

We employed a simple vectorial capacity model presented by Black & Moore [24] and as described by Kramer & Ebel [25]. This equation is based on the “basic reproductive rate” model described and developed by Macdonald [22, 23] and later modified by Garrett-Jones [26]. The model we employed estimates the mean number of infective bites arising per unit time from one infective host and is represented as:

$$VC=\frac{ma^{2}p^{n}b}{-\ln(p)}$$

Whereby *VC* = vectorial capacity; *m* = vector density in relation to the host; *a* = probability a vector feeds on a host in one day; *p* = probability of vectors surviving through one day; *n* = duration of the extrinsic incubation period (EIP) in days; *b* = vector competence (the proportion of vectors ingesting an infective meal that successfully become infective to another host), and 1/(-ln(*p*)) = duration of vector’s life in days after surviving the EIP (where ln denotes the natural logarithm).

### Estimation of parameters

Four species were chosen for consideration, based on implication in CHIKV transmission abroad and/or their association with humans and previous demonstration of vector competence in the laboratory [16]. The parameters used to estimate the vectorial capacity (VC) for each species were derived from previously published studies and unpublished data, with particular emphasis placed on observations from Australian populations, when such information was available.

Values for *m* (density) were identified as biting rates using an extrapolation from human landing collections which measure the number of attempted mosquito bites per human host over a defined period which, in our study, was per hour. The probability that a mosquito feeds on a host in one day (*a*) was calculated as the product of human host preference, feeding frequency and the number of multiple feeds comprising one replete bloodmeal. Host preference was identified from retrospective host feeding studies of bloodfed field-collected mosquitoes and defined as the proportion of bloodmeals identified from a human host (as opposed to meals from other animals). As no reservoir hosts, other than primates, have been identified for CHIKV, only the feeding rate on humans is applicable to the current study.

As per the Black and Moore definition of vectorial capacity adopted in this study [24], the feeding frequency is approximately equal to one divided by the length of the female reproductive cycle and was estimated from the duration of the gonotrophic cycle (in this case, the period of time between obtaining a bloodmeal and egg laying), after which a female mosquito would seek a subsequent bloodmeal. Vector competence (*b*) was based on transmission rates identified from laboratory infection studies using a Reunion Island isolate containing the alanine to valine amino acid substitution in the E1 envelope glycoprotein (E1:A226V) [16, 17]. For consistency, we chose day 14 transmission estimates for *Ae*. *albopictus* as these were the only values available for the other species. The vectorial capacity was calculated using an EIP of 10 days for all species because a recent study using *Ae*. *albopictus* demonstrated that the transmission rate peaked on this day [17]. Notwithstanding the likely variability in EIP due to variation in CHIKV strains and temperature, we selected 10 days as the EIP for the purposes of comparison between vector species as this likely represents the average EIP rather than the shortest observed EIP which may bias comparison between species [27]. Finally, all survival estimates (*p*) were estimated from field mark-release-recapture studies of wild populations, and represent the probability that a mosquito would survive through one day.

Where considerable differences in estimates of density and/or human feeding rates were found for some species, we chose minimum and maximum values to parameterise the model for comparison within those species.

For the purposes of comparison, it may be assumed that the vectorial capacity values reported here are valid for conditions of 28°C. Vector competence experiments for CHIKV [16, 17] were conducted at this temperature, and many field observations of host landing rates and daily survival are made during the summer months when temperatures are typically in the high 20s°C in Australian cities.

## Results

*Aedes aegypti*, *Ae*. *albopictus*, *Ae*. *notoscriptus* and *Ae*. *vigilax* were chosen for calculation of VC based on their association with humans, and demonstrated vector competence in the laboratory. The values identified from the literature and used to parameterise the model for each species are listed in Table 1, along with their sources. All parameter estimates pertaining to endemic species *Ae*. *notoscriptus* and *Ae*. *vigilax* were sourced from Australian studies, whereas estimates of parameters comprising *a* and *p* for *Ae*. *aegypti* and *Ae*. *albopictus* were, where necessary, sourced from studies conducted on mosquito populations from locations other than Australia (Table 1). The length of the gonotrophic cycle was designated as three days for all species, as these data were not available for all species, but was consistently identified as three days in most cases. Importantly, we acknowledge that these parameters are, to some extent, temperature-dependent, and the values used here will be subject to the laboratory and field conditions at which data were collected in source studies.

**Table 1:** Parameters identified from the literature and used to estimate vectorial capacity of Australian mosquito species for chikungunya virus.

		Species			
Parameter		*Aedes aegypti*	*Aedes albopictus*	*Aedes notoscriptus*	*Aedes vigilax*
*m*	Density in relation to host(human biting rate per hour)	4 (Queensland Health, state government data)	Maximum 48 for Masig and Warraber Islands or minimum 3.6 for Thursday Island (Queensland Health, state government data)	4.8 (mean of Summer and Winter observations in Brisbane [28])	Minimum 8.2 [29] recorded in Townsville and maximum 550.1 recorded in Redcliffe [30]
*a* ^a^	Host preference	0.75 [31] or 0.95 [32] ^c^	0.20 ^c^ [33] or 0.96 ^c^ [34]	0.19 [35] or 0.50 [36]	0.14 [31] and [35]
	Length of gonotrophic cycle (days)	3 ^c^ [37, 38] and reviewed in [39]	3 ^c^ [40]	3 [41]	3 [42]
	Multiple meals	2.8 [43]^c^	Possible, but likely to be small ^d^ (value of 1 used for calculation)	No (value of 1 used for calculation)	No (value of 1 used for calculation)
*p*	Survival	0.885 [44]	0.801 ^c^ [40, 45]	0.780 [46]	0.760 [47, 48]
*b*	Transmission rate	0.64 [16]	0.60 [17]	0.20 [16]	0.76 [16]
*n*	EIP	10 days^b^ [16, 17]			

^a^ *a* is probability a vector feeds on a host in one day, and is calculated as the product of host preference, feeding frequency (1/length of gonotrophic cycle) and multiple meals [24, 43]

^b^ EIP designated at 10 days for all species to represent time of peak infectivity [17]

^c^ indicates parameter value obtained from studies conducted on mosquito populations from origins other than Australia

^d^ information regarding the number of bloodmeals comprising a replete meal is lacking for *Ae*. *albopictus* [40] and it is unlikely to be influential (when compared with *Ae*. *aegypti*) due to limited probability of resuming a bloodmeal on another human

The influence of multiple feeds was only considered for *Ae*. *aegypti* as this behaviour has not been described for either *Ae*. *vigilax* or *Ae*. *notoscriptus*. While *Ae*. *albopictus* may occasionally feed on more than one host to complete a blood meal [33], it is anticipated that this behaviour occurs in less than 20% of bloodmeals [40] and, even when it does occur, we consider the likelihood of resuming a blood meal on a human to be low due to more eclectic feeding behaviour (a propensity to feed on a range of host types other than humans) when compared with *Ae*. *aegypti*.

Minimum and maximum estimates of host feeding rates (host preference) are based on the lowest and highest records identified in the literature. For *Ae*. *albopictus*, the maximum human host feeding rate identified was 0.957 [34] and the minimum was 0.2 [33], while the maximum identified for *Ae*. *aegypti* was 0.95 [32] and minimum was 0.75 [31]. Importantly, no Australian records of blood feeding habits or host preference were available for *Ae*. *albopictus*. The maximum and minimum densities identified from the literature for *Ae*. *vigilax* were 550.07 [30] and 8.2 [29], respectively, and for *Ae*. *albopictus* 48 and 3.6 (Queensland Health, state government data), respectively.

### Overall Results

**Fig 1:**
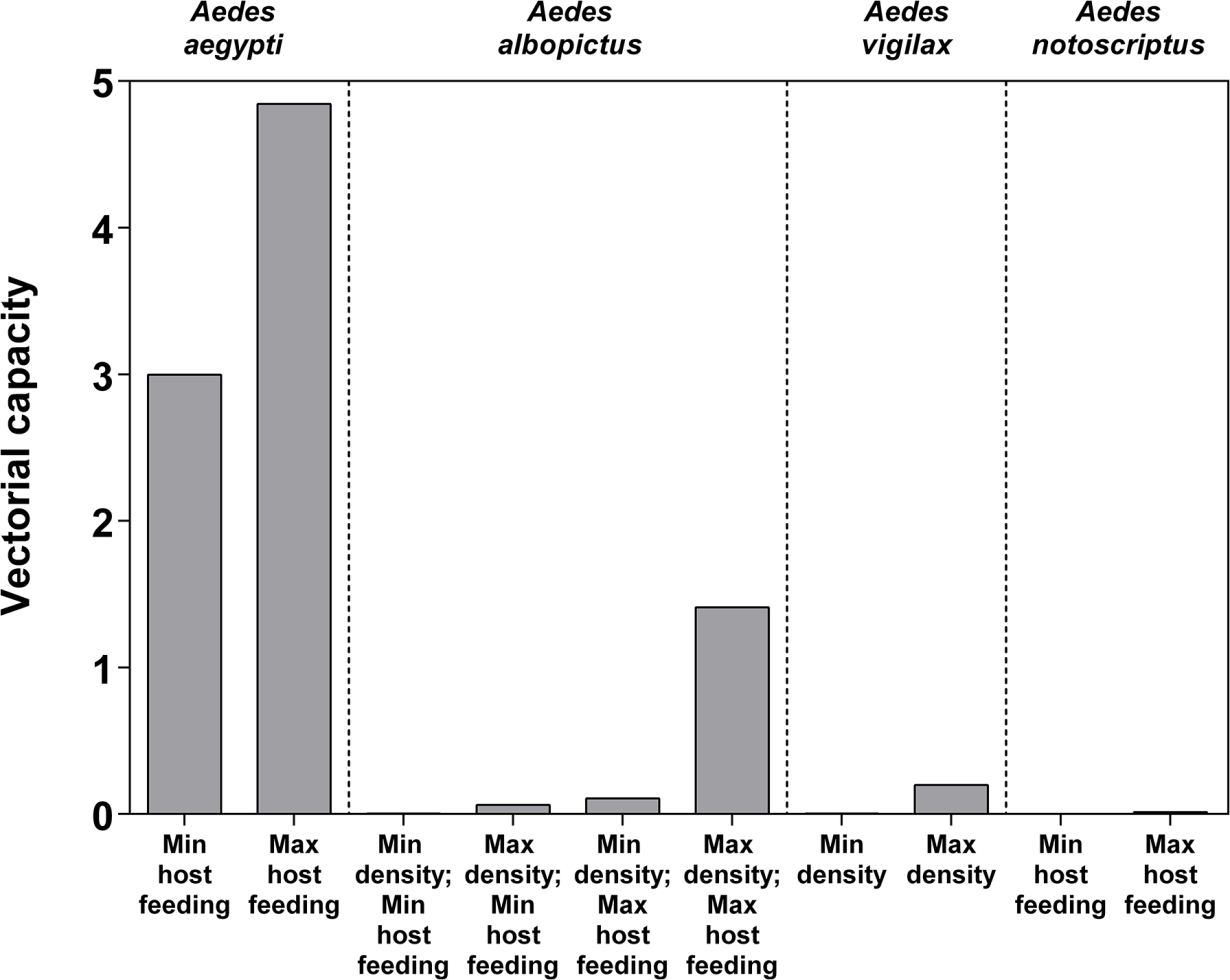
Comparison of vectorial capacity for four species, given maximum and minimum estimates of density and/or host feeding preferences where data available from the literature (refer Table 1).

When maximum and minimum human host feeding rates were employed in the model, *Ae*. *aegypti* demonstrated a VC of 3.00 and 4.85, respectively, which was considerably greater than that of *Ae*. *notoscriptus* and *Ae*. *vigilax* under all conditions, and that of *Ae*. *albopictus* under three of the four tested conditions (Fig 1). Even with minimum host feeding rates employed in the model, *Ae*. *aegypti* consistently demonstrated a much higher VC than all of the other species, under all conditions. When entered into the model with maximum density and human host feeding rate estimates, *Ae*. *albopictus* demonstrated a VC of 1.44, which was the highest estimate of the remaining species. However, when minimum density and/or host feeding rates were used, VC values for *Ae*. *albopictus* were consistently less than 0.15. Values of VC calculated for both *Ae*. *vigilax* and *Ae*. *notoscriptus* were less than 0.22 under all conditions. Even with a maximum density estimate of over 500 bites per person per day (the greatest estimate for any of the species considered), the VC of *Ae*. *vigilax* was 75% less than that of *Ae*. *aegypti*. Similarly, even at the maximum human host feeding rate, *Ae*. *notoscriptus* demonstrated a VC of < 0.02.

## Discussion

Preparedness for potential urban transmission of CHIKV in Australia is critical. A key element of a pre-emptive management plan is the incrimination of potential local vectors, with consideration of their vector competence together with biological and ecological attributes, which determine their relative roles in field transmission. This activity can enhance preparedness measures, in addition to reducing confusion when interpreting results from laboratory studies in a public health setting. Using a simple vectorial capacity model, we compared the relative potential roles of urban mosquito species in field transmission of CHIKV. We chose four species for consideration, based on laboratory vector competence experiments [16], widespread geographical distributions, association with urban habitats, and the relative abundance of these species in such locations.

Unsurprisingly, the model shows that *Ae*. *aegypti* is a key candidate vector of CHIKV in Australia regardless of whether minimum or maximum estimates of human feeding rates were employed, and suggests that *Ae*. *aegypti* would most likely be the primary potential Australian vector across most conditions. This species was the first species to be implicated in urban CHIKV transmission and, despite the incrimination of *Ae*. *albopictus* in a number of recent outbreaks (particularly since the virus mutation described by Schuffenecker et al. [49] was associated with enhanced infectivity in *Ae*. *albopictus*;[50]), *Ae*. *aegypti* remains an important vector in a number of locations [51, 52], and has been confirmed to be equally competent for the recently emerged strain of CHIKV [53]. The important role of *Ae*. *aegypti* in the transmission of pathogens that are maintained in a human-mosquito-human transmission cycle has been repeatedly demonstrated using similar models to that described herein [43, 54, 55]. Notably, as the probability of the vector feeding on a host in a day (*a*) appears as a squared element in the model, the VC is very sensitive to changes in the values for *a*. Thus, consistent anthropophilly throughout its urban distribution, combined with the propensity for *Ae*. *aegypti* to obtain multiple bloodmeals in a replete meal (two unique features amongst the species considered herein), can explain why *Ae*. *aegypti* is consistently implicated as the most important candidate vector in the current study under all conditions.

Currently, the geographical distribution of *Ae*. *aegypti* in Australia is restricted to northern coastal areas and some townships of central and southern Queensland. In Northern Queensland *Ae*. *aegypti* periodically transmits dengue viruses, following the importation of virus by travellers that have acquired the infection overseas. In a similar way, it is certainly feasible that *Ae*. *aegypti* could be a likely candidate vector of imported CHIKV throughout its geographic distribution, and these regions should consider appropriate control strategies.

Vectorial capacity changes are most sensitive to the daily survival probability of the vector (*p*), and the extrinsic incubation rate of the virus (*n*) [24]. Furthermore, changes to CHIKV characteristics that impact EIP will influence VC of *Ae*. *aegypti*. Christofferson and others [27] showed the effect of EIP on transmission potential was much greater than the effect of vector competence and that divergent strains of CHIKV vary in terms of the observed EIP between and within vector species. Similarly, even slight increases in daily mosquito survival can lead to disproportionately large increases in VC. Thus, if CHIKV were to become established in Australia, it would immediately be critical to examine both the EIP in local populations and confirm the typical survival rates of candidate species under local conditions as a matter of priority.

Using estimates of maximum density and maximum human host feeding rate, *Ae*. *albopictus* was also shown to be a potential Australian candidate vector for CHIKV, with VC estimates around half that calculated for *Ae*. *aegypti* under its conservative (minimum estimate) conditions. This suggests that *Ae*. *albopictus* could present a significant risk in urban areas where alternative hosts may be scarce, and if domestic habitats are abundant and productive. Currently, the geographical distribution of *Ae*. *albopictus* is restricted to the Torres Strait region, but there is considerable concern that this species may establish on mainland Australia [21, 56, 57]. The Torres Strait should be considered a high risk location for potential CHIKV transmission due to the presence of both *Ae*. *albopictus* and *Ae*. *aegypti*, in addition to its close proximity to PNG which has recently experienced explosive CHIKV activity [12]. Indeed, PNG was the source of the majority of imported chikungunya cases in Queensland during the outbreak (11 of 14 cases reported in Queensland in 2013; D. Francis, Queensland Health, pers. comm.). Importantly, it must also be recognised that management of vector borne diseases in Torres Strait region is particularly challenging due to the relative remoteness of the region and a lack of data describing behavioural characteristics and population dynamics of local *Ae*. *albopictus* populations, as this species was only detected in the Torres Strait relatively recently [21]. Our analysis suggests that when at high densities, *Ae*. *albopictus* may be an important vector of CHIKV, provided that humans are the predominant source of bloodmeals. As observed for *Ae*. *aegypti*, the human host feeding rate can dramatically influence their projected VC, and it is noteworthy that in the current study estimates of human feeding rates of *Ae*. *albopictus* were sourced from studies conducted abroad, as host feeding patterns of Australian populations have not yet been examined. Furthermore, should multiple feeding on different human hosts by *Ae*. *albopictus* occur, this would increase VC strongly. If field data demonstrating this were available, recalculated VC for this species would likely approach that calculated for *Ae*. *aegypti*. Overall, the implication of *Ae*. *albopictus* as a potential vector of CHIKV in Australia underscores the urgent need to obtain host feeding data describing the behaviour of Australian populations of *Ae*. *albopictus* to further resolve the potential role of this species in CHIKV transmission.

In addition to human feeding rates, density was also shown to be a key determinant of estimated VC for *Ae*. *albopictus* by the current model. Importantly, the density of *Ae*. *albopictus* varies considerably between islands of the Torres Strait and the current study underscores the importance of understanding such variation across islands to support decision making on vector control with respect to CHIKV transmission risk. Further, should *Ae*. *albopictus* manage to establish in southern regions on the Australian mainland [58, 59], the VC of *Ae*. *albopictus* for CHIKV transmission in temperate Australia would need to be re-evaluated cognisant of the temperature-dependent nature of CHIKV EIP and mosquito biology and ecology.

Confirmation of the relative importance of *Ae*. *aegypti* and *Ae*. *albopictus* in the potential transmission of CHIKV in Australia demonstrated here highlights the value of efforts to prevent these species from expanding their geographical ranges and underscores the importance of targeted surveillance activities to provide early detection of the incursion of these vectors in novel locations. This is of particular importance to urban population centres, particularly those which experience high numbers of imported case notifications. Should populations of *Ae*. *aegypti* or *Ae*. *albopictus* become established in such urban centres on the Australian mainland, it would represent a significant risk of explosive CHIKV transmission, potentially placing a considerable proportion of the human population at risk of infection.

The remaining species considered herein are likely to play a relatively minor role in potential CHIKV transmission, when compared with either *Ae*. *aegypti* or *Ae*. *albopictus*. For example, despite the ability of *Ae*. *vigilax* to reach very high population densities particularly after high tides during humid conditions, this species would likely play a limited role in transmission, according to the current model. Nevertheless, at the highest population density considered, this species would likely play a larger role in urban transmission of CHIKV than *Ae*. *notoscriptus* despite the tendency for *Ae*. *notoscriptus* to exploit backyard habitats. Indeed, the model suggests that *Ae*. *notoscriptus* would have a minimal role in the potential transmission of CHIKV in Australia. However, recent genetic data suggests that *Ae*. *notoscriptus* may comprise at least three species in Australia [60], so geographical differences in behaviour, ecology and biology should be further considered. However, the current model suggests that neither *Ae*. *vigilax* nor *Ae*. *notoscriptus* alone would likely maintain large and explosive outbreaks similar to those observed elsewhere in recent years. This may be viewed as propitious, given the widespread geographical distribution of these two species across all states of Australia, including prevalence in capital cities, where the majority of imported cases of CHIKV are reported.

Notwithstanding, a number of other factors would likely contribute to the likelihood and magnitude of a CHIKV outbreak in Australia. Of these, the number of imported cases in a given region is arguably among the most important. Imported cases represent an opportunity for local transmission if the case is viraemic and suitable vectors are present. Imported cases of CHIKV have been recorded in all Australian states and mainland territories (with the exception of the Australian Capital Territory where CHIKV is not considered a notifiable condition), with the majority of cases reported in the most populous states of New South Wales, Victoria, Queensland and Western Australia [15]. Of these, Queensland is the only state with established populations of *Ae*. *aegypti* and/or *Ae*. *albopictus*. Importantly, the Cairns local government area (the location of the majority of local dengue transmission) has recorded the second highest number of total CHIKV importations between 2010–2014 in Queensland, outnumbered only by the Brisbane metropolitan region, highlighting the potential vulnerability of northern Queensland where these candidate vectors are present [61].

Importantly, the strain of virus can determine the likelihood of local transmission, and the potential magnitude of ensuing local transmission. Molecular characterization of an Indian Ocean CHIKV strain revealed that the E1:A226V mutation led to increased replication and transmission efficiency in *Ae*. *albopictus* which likely contributed to the scale of the Indian Ocean epidemic [50, 53, 62]. Subsequent substitutions in the E2 envelope glycoprotein in some virus populations have further enhanced CHIKV fitness in *Ae*. *albopictus* [63, 64]. Interestingly, these substitutions did not confer a fitness advantage in *Ae*. *aegypti*. In another study, Vega-Rua et al. [65] demonstrated considerable variation within and between North and South American populations of *Ae*. *aegypti* and *Ae*. *albopictus* in their ability to become infected with and transmit strains of CHIKV with and without the E1:A226V mutation. The data we used to estimate transmission rates was sourced from vector competence experiments conducted using a Reunion Island isolate containing the E1:A226V mutation [16, 17], and is the only strain that has been characterized in Australian mosquitoes. Given the emergence of the Asian lineage of CHIKV in the Americas and some locations in the Pacific, vector competence experiments are urgently required to assess the ability of Australian mosquito populations to transmit strains of CHIKV which do not contain the E1:A226V mutation. The recalculated VC of incriminated species for other strains of CHIKV should be used to assess the potential for outbreaks caused by different CHIKV lineages.

Australia possesses a number of unique attributes which may impact the likelihood of CHIKV causing explosive transmission similar to that which has occurred in the Indian Ocean region and the Americas. Importantly, CHIKV is antigenically related to endemic Ross River virus (RRV), which circulates in an enzootic cycle in all mainland states and is responsible for the majority of arbovirus disease notifications in Australia [15, 66]. Given that marsupials are the primary hosts of RRV [66], it is possible that marsupials could develop sufficient CHIKV viraemia to infect mosquitoes. Anti-RRV antibodies can reduce the viraemia and severity of disease associated with CHIKV infection in mice [67], so it is plausible that humans with prior infection with RRV may be protected against CHIKV infection and associated disease. Concomitantly, a reduction in viraemia levels could reduce the pool of available hosts capable of infecting recipient mosquitoes and perpetuating transmission. However, the 2012–2013 epidemic in PNG (where RRV is likewise endemic) suggests that whilst prior exposure to RRV may modulate CHIKV infection, it will not prevent transmission.

Public health professionals need to exercise caution when interpreting the results from laboratory vector competence studies, particularly when inferring the potential role of endemic mosquito species in potential field transmission of exotic viruses and developing pre-emptive management strategies. The vectorial capacity model is a tool to aid the understanding of the relative potential importance of vector species in the transmission of a given pathogen, and provides a relative quantitative index of a mosquito population’s capacity to transmit the pathogen to a susceptible host population. A vectorial capacity model can also highlight key biological and ecological factors which require further characterisation to underpin informed risk assessment. Importantly, this model can be applied to assess the role of endemic species in the transmission of other viruses of public health concern to Australia, including the recently emergent Zika virus [68, 69].

When applied to candidate Australian vectors of CHIKV, the VC model suggests that *Ae*. *aegypti* and *Ae*. *albopictus* are, overwhelmingly, the most likely species to sustain local transmission of CHIKV. Secondary vector species are likely to be able to maintain local transmission, but unlikely to sustain outbreaks of an explosive nature as observed in recent outbreaks abroad. Nevertheless, their potential role in urban transmission of CHIKV cannot be discounted and the biological and behavioural characteristics of local populations must be considered when developing control strategies or management plans. Although our study focussed on the Australian context, the relatively simple analysis presented herein has application to other regions of the world where vector competence experiments have implicated potential vectors, but for which there is limited information on their actual role in CHIKV transmission cycles.
